# MicroRNA-138 is a potential regulator of memory performance in humans

**DOI:** 10.3389/fnhum.2014.00501

**Published:** 2014-07-11

**Authors:** Julia Schröder, Sara Ansaloni, Marcel Schilling, Tian Liu, Josefine Radke, Marian Jaedicke, Brit-Maren M. Schjeide, Andriy Mashychev, Christina Tegeler, Helena Radbruch, Goran Papenberg, Sandra Düzel, Ilja Demuth, Nina Bucholtz, Ulman Lindenberger, Shu-Chen Li, Elisabeth Steinhagen-Thiessen, Christina M. Lill, Lars Bertram

**Affiliations:** ^1^Department of Vertebrate Genomics, Max Planck Institute for Molecular GeneticsBerlin, Germany; ^2^Charité Research Group on Geriatrics, Charité – Universitätsmedizin BerlinBerlin, Germany; ^3^Max Delbrück Center for Molecular Medicine, Berlin Institute for Medical Systems BiologyBerlin, Germany; ^4^Center for Lifespan Psychology, Max Planck Institute for Human DevelopmentBerlin, Germany; ^5^Department of Neuropathology, Charité – Universitätsmedizin BerlinBerlin, Germany; ^6^Aging Research Center, Karolinska InstituteStockholm, Sweden; ^7^Institute of Medical and Human Genetics, Charité – Universitätsmedizin BerlinBerlin, Germany; ^8^Department of Psychology, Lifespan Developmental Neuroscience, TU DresdenDresden, Germany; ^9^Focus Program Translational Neuroscience, Department of Neurology, University Medical Center of the Johannes Gutenberg University MainzMainz, Germany; ^10^Faculty of Medicine, School of Public Health, Imperial College LondonLondon, UK

**Keywords:** genome-wide association study, GWAS, working memory, episodic memory, microRNA, hsa-mir-138-5p, *DCP1B*

## Abstract

Genetic factors underlie a substantial proportion of individual differences in cognitive functions in humans, including processes related to episodic and working memory. While genetic association studies have proposed several candidate “memory genes,” these currently explain only a minor fraction of the phenotypic variance. Here, we performed genome-wide screening on 13 episodic and working memory phenotypes in 1318 participants of the Berlin Aging Study II aged 60 years or older. The analyses highlight a number of novel single nucleotide polymorphisms (SNPs) associated with memory performance, including one located in a putative regulatory region of microRNA (miRNA) hsa-mir-138-5p (rs9882688, *P*-value = 7.8 × 10^−9^). Expression quantitative trait locus analyses on next-generation RNA-sequencing data revealed that rs9882688 genotypes show a significant correlation with the expression levels of this miRNA in 309 human lymphoblastoid cell lines (*P*-value = 5 × 10^−4^). *In silico* modeling of other top-ranking GWAS signals identified an additional memory-associated SNP in the 3′ untranslated region (3′ UTR) of *DCP1B*, a gene encoding a core component of the mRNA decapping complex in humans, predicted to interfere with hsa-mir-138-5p binding. This prediction was confirmed *in vitro* by luciferase assays showing differential binding of hsa-mir-138-5p to 3′ UTR reporter constructs in two human cell lines (HEK293: *P*-value = 0.0470; SH-SY5Y: *P*-value = 0.0866). Finally, expression profiling of hsa-mir-138-5p and *DCP1B* mRNA in human post-mortem brain tissue revealed that both molecules are expressed simultaneously in frontal cortex and hippocampus, suggesting that the proposed interaction between hsa-mir-138-5p and *DCP1B* may also take place *in vivo*. In summary, by combining unbiased genome-wide screening with extensive *in silico* modeling, *in vitro* functional assays, and gene expression profiling, our study identified miRNA-138 as a potential molecular regulator of human memory function.

## Introduction

Interindividual variations of memory performance in humans are regulated by genetic and non-genetic factors. Early estimates from twin studies suggest that approximately half of the phenotypic variance is attributable to heritable factors, while the remainder reflects shared and non-shared environmental factors (McClearn et al., 1997). These estimates have since received broad support from studies using different designs and analysis approaches (for recent review see Goldberg Hermo et al., 2014). For measures of general cognitive ability, the presumed genetic effects appear to increase across the lifespan, that is, from childhood to adulthood and late life (McClearn et al., 1997), suggesting that searching for genes in this domain may be most powerful in data sets of aged individuals (see also Lindenberger et al., 2008).

A number of candidate genes that may affect various aspects of memory performance in humans have been proposed to date (for review see Papassotiropoulos and de Quervain, 2011). As is the case for many other genetically complex traits in humans, the most convincing of these have only recently emerged in the context of genome-wide association studies (GWAS; see NHGRI's “GWAS catalog” for an up-to-date overview; URL: http://www.genome.gov/gwastudies/; Welter et al., 2014). Among the most prominent findings are common polymorphisms (i.e., single nucleotide polymorphisms [SNPs]) in *WWC1* (WW and C2 containing 1, a.k.a. *KIBRA* for kidney and brain expressed protein; Papassotiropoulos et al., 2006) and *CTNNBL1* (catenin, beta like 1). *WWC1* is located on chromosome (chr) 5q34 and was identified nearly a decade ago in a GWAS on episodic memory in ~300 subjects in which the lead SNP (rs17070145) showed evidence for genome-wide significant association (Papassotiropoulos et al., 2006). Since the original study, a number of follow-up studies have been published, albeit with mixed results (Milnik et al., 2012). The other lead “memory gene,” *CTNNBL1* (located on chr 20q11.23) was identified by the same group in a more recent GWAS (Papassotiropoulos and de Quervain, 2011). Thus, far, no reports have been published confirming this latter finding independently. In addition, a number of other candidate genes have been tested in non-GWAS association studies, some suggesting evidence for an increased effect sizes when comparing older vs. younger adults (Li et al., 2013; Papenberg et al., 2014).

A second—and thus far largely independent—line of genetic experiments suggests that memory performance, and likely a large number of other cognitive domains, may be influenced by the action of microRNAs (miRNAs). MiRNAs are short (i.e., typically between 18 and 24 nucleotide long), non-coding RNA molecules that are involved in regulating protein expression post-transcriptionally. This is achieved by binding to the target messenger-RNAs (mRNAs), thereby directly or indirectly interfering with mRNA translation. The last decade of research has shown that miRNAs are involved in a broad range of cellular functions, including the development, differentiation, proliferation, apoptosis, and metabolism of neurons and many other cell types in humans (Satoh, 2012). Some estimates suggest that the expression of up to 50% of all human proteins may be affected by the action of one or more of the >2500 miRNAs currently believed to exist (URL: http://mirbase.org/index.shtml; Griffiths-Jones et al., 2006). One important factor determining the extent of miRNA-mediated expressional regulation is the binding affinity between miRNAs and their target mRNAs. This is largely influenced by sequence complementarity between the respective interacting regions on both molecules (Peterson et al., 2014). Naturally occurring DNA sequence variants, e.g., trait associated SNPs, within the binding domains of either of these interactants can thus be expected to interfere with miRNA-to-mRNA binding either by decreasing (disruption of complementary sites) or increasing (creation of complementary sites) binding affinity.

In this study, we specifically searched for memory-associated DNA sequence variants predicted to affect miRNA-to-mRNA binding using a similar strategy as described previously by our group (Lill et al., 2014). Genetic associations, with measures of both working and episodic memory functions were assessed via genome-wide screening as part of an ongoing GWAS in participants of the Berlin Aging Study II (BASE-II). Associated SNPs were evaluated for their potential effects on miRNA-to-mRNA binding *in silico* using a bioinformatic prediction tool developed by our group. SNPs showing association with memory performance and predicted to directly alter miRNA-to-mRNA binding were further followed up using a range of *in vitro* experiments involving luciferase reporter assays in two human cell lines, as well as miRNA and mRNA expression profiling in human brain autopsy material from three adult individuals. Our analyses uncovered three memory-associated SNPs which potentially manifest their molecular effects by interfering with miRNA function. Intriguingly, two of these SNPs, by independent mechanisms, affect hsa-mir-138-1, a miRNA long known to be crucial in CNS development and function in mammals (Miska et al., 2004; Siegel et al., 2009) but hitherto not specifically linked to cognitive performance in humans.

## Methods

### Genome-wide association study (GWAS) of episodic and working memory performance in humans

#### Participants

All GWAS participants were part of the Berlin Aging Study II (BASE-II), a multidisciplinary project investigating factors involved in “healthy” vs. “unhealthy” aging (Bertram et al., 2013). In addition to genetics, BASE-II covers a broad range of functional domains critical for understanding aging investigated in a multidisciplinary assessment protocol that includes measures from internal medicine, immunology, psychology, as well as sociology and economics. The behavioral test battery applied to each BASE-II participant includes an extensive coverage of cognitive abilities, including detailed assessments of working and episodic memory. At baseline, BASE-II includes 2200 participants of Caucasian ancestry recruited from the greater Berlin area. The cohort is split into a subgroup of 1600 older adults aged 60–80 years, mean 66.76 years at baseline, and 600 younger adults aged 20–35 years, mean 27.32 years. Both groups consist of equal numbers of males and females. The analyses presented here are limited to participants of the “old” stratum for whom genotype and cognitive data were available at the time of analysis (*n* = 1318). Written consent was provided by all BASE-II individuals before participation. The study was approved by the institutional review boards of each relevant participating research unit prior to participant recruitment.

#### Assessment of memory performance

This study is based on 13 different quantitative measures (i.e., 2 and 11, respectively) of working memory (WM) and episodic memory (EM) were selected, assessed either at the Center for Lifespan Psychology at the Max Planck Institute for Human Development (MPIHD; *n* = 1318 individuals with cognitive testing completed at time of analysis) or at the Charité Research Group on Geriatrics (CRGG; *n* = 961 with cognitive testing completed at time of analysis) at Charité University Hospital. At MPIHD, six participants from the same age group were tested simultaneously in two separate group sessions 1 week apart. In addition, we utilized test results from the CERAD Plus battery (Morris et al., 1989; Fillenbaum et al., 2008), which was carried out individually to each participant at CRGG. See Supplementary Table 1 and Supplementary Methods for more detailed information on the type of WM and EM assessments used here.

#### Genotyping and genetic association analyses

Details on genotyping and analysis procedures can be found in the Supplementary Methods. In brief, DNA from all BASE-II participants was extracted from whole blood using standard procedures and then subjected to microarray-based SNP genotyping using the “Genome-Wide Human SNP Array 6.0” (Affymetrix, Inc.), followed by an extensive quality control and genome-wide imputation of unobserved genotypes using whole genome sequence data from the 1000 Genomes Project (Abecasis et al., 2010). Association analyses were carried out using the EM or WM variables as quantitative traits assuming an additive linear model, adjusted for age, sex, and years of education and to the first three principal components to account for potential population stratification. Association analyses were performed using SNPTEST v.1.3 (Marchini and Howie, 2010), which accounts for uncertainty of imputed genotype calls via missing data likelihood tests. Overall, in this study we tested a total of 12,607,232 high-quality SNPs for genetic association with the memory traits in 1318 (MPIHD) and 961 (CRGG) subjects from the BASE-II subgroup aged 60–80 years.

### *In silico* predictions of miRNA-to-mRNA binding and potential SNP effects

To systematically assess the potential impact of SNP allele-status on miRNA-to-mRNA binding, we utilized a bioinformatic tool recently developed by our group described in detail elsewhere (Schilling, 2011, 2013). In brief, this entailed a prediction of potential miRNA binding sites for 3′UTRs of all known protein-coding transcripts (downloaded from Ensembl Genes 71, http://www.ensembl.org/biomart/martview) using miRanda v.3.38, TargetScan 5.09 and PITA and v19 of the mirBASE database (http://www.mirbase.org). Only SNPs displaying strong linkage disequilibrium (LD; i.e., *r*^2^ of 0.8; estimated from whole genome sequence data of the 1000 Genomes phase 1 CEU reference panel; Abecasis et al., 2010) with GWAS SNPs were considered further. For these SNPs, we finally estimated the potential effects on miRNA-to-mRNA binding using a modified version of the support vector regression (SVR) method developed by Betel (Betel et al., 2010).

### *In vitro* assessment of SNPs predicted to affect miRNA-to-mRNA binding

#### Cell culture and construct transfection

Experiments were performed as previously described (Lill et al., 2014). Custom made reporter plasmids (pLightSwitch_3UTR) containing the appropriate 3′UTR sequence and the miRNA mimics were purchased from SwitchGear Genomics (Menlo Park, CA, USA). The desired 3′UTRs were subcloned in the pLightSwitch_3UTR vector downstream of the *Renilla* luciferase gene. The UTR constructs containing the reference allele were used as a template to generate point mutations via site directed mutagenesis. All constructs were verified by Sanger sequencing. 3′UTR constructs were transfected into naïve human embryonic kidney (HEK293) and human neuroblastoma (SH-SY5Y) cells, which were cultured in DMEM GlutaMax (Invitrogen, Darmstadt, Germany) media with 10% FBS (Biochrom, Berlin, Germany) for HEK293 or 15% FBS for SH-SY5Y cells, with an additional 1% Penicillin/Streptomycin (Biochrom). The cells were grown in standard conditions (37°C, 5% CO_2_). Transfection was carried out in 96-well plates (TPP, Trasadingen, Switzerland) at a cell confluency of about 50% using Dharmafect (ThermoScientific) following manufacturer's instructions. 50 ng of vector with the desired 3′UTR and 50 nM of corresponding miRNA mimic or scrambled non-binding miRNA were co-transfected in the cells per well. The scrambled miRNA was used as a negative control in combination with all 3′UTR constructs and in all experiments. After 24 h, HEK293 and SH-SY5Y cells were collected by freezing the culture plates directly on dry ice to enhance cell lysis. The plates were then thawed on ice and the resuspended cell lysates used for the luciferase assays. The LightSwitch Assay reagents (SwitchGear Genomics) were used following manufacturer's instructions. Assay reagents mixed with the same volume of cell lysate were transferred to a white 96-well plate (Costar, Washington, D.C., USA). An end point read of *Renilla* luciferase intensity values was taken using the POLARStar Omega (BMG Labtech, Ortenburg, Germany) plate reader with 3 s integration time and 3500 gain per well. Five to seven independent experiments per cell line and experimental condition were performed, using independent transfection mixes and/or different cell batches. For each independent experiment, six replicates were performed.

#### Statistical analysis of luciferase data

The analysis of the luciferase assay results were performed using R, an open-source language and environment for statistical computing and graphic (URL: http://www.r-project.org). We observed no outliers defined as deviating more than three standard deviations from the mean luciferase luminescence per experimental condition in each independent experiment. For each independent experiment, the mean luciferase activity of the 3′UTR reporter construct (i.e., containing either the reference or the alternative allele) co-transfected with a functional miRNA was divided by the baseline mean luciferase activity of the corresponding reporter construct co-transfected with the scrambled, non-targeting miRNA as negative control. To assess binding of the miRNAs to their predicted target sites irrespective of allele status, normalized luciferase activity of either 3′UTR reporter construct (i.e., either containing the reference or the alternative allele) co-transfected with the functional miRNA was compared to the control condition using the non-binding miRNA by one-sample *t*-test (*P*-values reported for this analysis are one-tailed). Changes in *Renilla* gene expression levels in 3′UTR constructs containing the reference vs. alternative alleles were assessed based on the *t*-test statistic for two independent samples (*P*-values reported for this analysis are two-tailed).

### Analysis of expression levels of miRNA and mRNA molecules in human post-mortem brain tissue

#### Tissue preparation and RNA extraction

Human brain tissue was collected post-mortem from hippocampi and frontal cortices from three deceased individuals without history of neuropsychiatric diseases at the Charité University Hospital (Berlin, Germany). After collection, brain samples were stored (at −20°C) in RNA*later*® solution (Applied Biosystems, Forster City, CA, USA) to avoid degradation. We used the miRVANA™ miRNA Isolation Kit (Life Technologies, Darmstadt, Germany) to extract small and total RNAs from tissue samples following manufacturer's instructions. Prior to extraction, all samples were homogenized using TissueLyser (QIAgen, Hilden, Germany) by shaking each sample twice in Lysis/Binding buffer for 2 min at 20 Hz.

#### Assessment of miRNA expression levels

The quantification procedure comprises two steps: reverse transcription from RNA to cDNA followed by the amplification via quantitative PCR (qPCR). Specific primers for reverse transcription and qPCR were based on pre-made TaqMan® Small RNA Assays (Applied Biosystems). Reverse transcription was performed on 10 ng RNA using the Taqman® MiRNA Reverse Transcription Kit according to the manufacturer's protocol (Applied Biosystems). The qPCR reaction was conducted using TaqMan® Small RNA Assays following the manufacturer's protocol (Applied Biosystems). In short this entailed: For reverse transcription 10 ng of the extracted RNA was used in a reaction mix containing Reverse Transcription Buffer, 15 mM dNTPs, 50 U/μl MultiScribe™ Reverse Transcriptase, 20 U/μl RNase Inhibitor, and Primer in a final volume of 15 μl. The protocol in 384-well format for all reactions was as follows: 16°C for 30 min, 30 min at 42°C and a final step of 85°C for 5 min. The qPCR reaction was conducted in TaqMan® Universal PCR Master Mix II, TaqMan® Small RNA Assay (both Applied Biosystems) and 1.33 μl of the RT-PCR product in a final volume of 20 μl. The cycling protocol was: 10 min at 95°C, 15 s at 95°C, 60 s at 60°C for overall 50 cycles. The reactions were run and visualized on a QuantStudio™ 12 K Flex Real-Time PCR System (Applied Biosystems).

#### Assessment of mRNA expression levels

Reverse transcription was performed using the High Capacity RNA-to-cDNA Kit (Applied Biosystems) on 0.2 μg of total RNA following the manufacturer's instructions. The reactions were run on the Thermo Cycler PTC-240 (MJ Research, Waltham, MA, USA). Expression of the resulting *DCP1B* cDNA was assessed via PCR primers 5′-CCAGGGTCTCCTCACAACAT-3′ (forward) and 5′-TCTTTTTCATGGCTGCTTGA-3′ (reverse). Primers were designed to lead to a ~850 bp cDNA amplicon vs. a 6.9 kb gDNA amplicon. PCR conditions were using 1.5 μM of each primer, approximately 60 ng of cDNA template, 0.25 nM dNTPs, 10 mM MgCl_2_, 30% Q solution (QIAgen) and 0.25 U Taq polymerase in a final volume of 10 μl. Reactions were carried out in 96-well format on PTC-240 thermal cyclers at 94°C (3 min), followed by 40 cycles of 94°C (45 s), 60.5°C (90 s), and 72°C (60 s), and a final extension step of 72°C (6 min). PCR amplicons were visualized by ethidium bromide stained gel electrophoresis in 1% agarose (Sigma Aldrich, Taufkirchen, Germany).

### Expression quantitative trait locus (eQTL) analyses of hsa-miR-138-5p using next-generation sequencing data

eQTL analyses on the potential role of rs9882688 in expression of hsa-miR-138-5p were performed using next-generation small RNA sequencing data of peripheral lymphoblastoid cell line (LCL) samples generated by the Genetic European Variation in Health and Disease (GEUVADIS) consortium (for a description of sequencing methods and data preparation, see Lappalainen et al., 2013). For the eQTL analyses here, we downloaded normalized expression data of hsa-miR-138-5p for four populations of European descent [i.e., Utah Residents with Northern and Western European Ancestry (CEU), Finns (FIN), British (GBR), Toscani (TSI)] as released by the GEUVADIS project database (URL: http://www.ebi.ac.uk/arrayexpress/files/E-GEUV-2/GD452.MirnaQuantCount.1.2N.50FN. samplename.resk10.txt). Subject-level genotypes for rs9882688 in the same individuals were obtained from the 1000 Genomes database (URL: http://browser.1000genomes.org/index.html; Abecasis et al., 2010). The resulting data set included 333 individuals of European descent with both hsa-miR-138-5p expression data and rs9882688 genotypes. Statistical analyses of these data were performed using R. MiRNA expression values that fell 1.5× the interquartile range (IQR) below the first quartile or 1.5× the IQR above the third quartile were defined as outliers and excluded from further analysis (24 samples). Expression levels of hsa-miR-138-5p showed a symmetrical, approximately normal distribution in the effective sample size of 309 individuals [as determined by the Shapiro–Wilk test implemented in R (*P* = 0.548) and quantile–quantile plotting, Supplementary Figure 1]. Subsequent eQTL analyses on these 309 samples were based on an additive model using linear regression. Association results were adjusted for sex and population of origin. Due to the low frequency of homozygote carriers of the G allele (*n* = 2), sensitivity analyses were performed upon exclusion of those individuals. Statistical significance for these analyses is expressed as two-tailed *P*-values.

## Results

### GWAS of episodic and working memory performance in humans

The GWAS analyses on 13 episodic and working memory traits in up to 1318 individuals from the subgroup of BASE-II participants aged 60 years or older revealed 28 distinct genomic regions (or: loci) showing association *P*-values at 1 × 10^−6^ or below (Supplementary Table 2), indicating that at least some of these are genetically linked to human memory performance. Notably, these did not include SNPs in *WWC1* (*KIBRA*) or *CTNNBL1* (Liu et al., in press), suggesting that the analyzed traits are not significantly influenced by SNPs in these genes in our study population. The three most significant findings showed *P*-values at or below 1 × 10^−7^ and were observed with SNPs rs9882688 (*P* = 7.8 × 10^−9^ on chr 3p21.32) for trait “WL_save” (part of the CERAD cognitive battery measuring the proportion of learned words vs. recalled words), with rs1016365 (*P* = 9.7 × 10^−8^ on chr 8q13.3) for “ItemItem” (an associative episodic memory task where probands were asked to learn and recall words irrespective of their pairing with other words), and with rs113948889 (*P* = 9.9 × 10^−8^ on chr 12p13.33) for “TFEUWC” (a spatial working memory paradigm combined with tasks testing frontal executive control; see Supplementary Material for more information on these and all remaining traits). All of these signals were flanked by multiple additional SNPs showing *P*-values at or below 1 × 10^−5^ indicating that they do not reflect technical artifacts (see Supplementary Figures 2–4 for Manhattan and Q–Q plots for GWAS results of these three traits). Only the signal with rs9882688 on chr 3 surpassed the threshold for genome-wide significance (i.e., *P*-values at or below 5 × 10^−8^, a cutoff frequently used in the context of GWAS, see McCarthy et al., 2008). This SNP, which itself does not map to any known open reading frame, is located approx. 20 kb upstream from the 5′ end of miRNA hsa-mir-138-1 and, thus, potentially within active upstream regulatory elements of this miRNA. This interpretation is supported by the fact that rs9882688 is located within an ENCODE DNase I hypersensitivity cluster and within a known H3K27Ac mark, two features typically characterizing active regulatory elements (ENCODE Project Consortium et al., 2012). The second leading GWAS signal was elicited by SNP rs1016365, which is located approx. 6 kb 3′ of *EYA1* (Homo sapiens eyes absent homolog 1 [Drosophila]) a member of the eyes absent (EYA) family of proteins. The third best associated SNP in our GWAS (rs113948889) maps to an intron of *DCP1B* (encoding decapping mRNA 1B), which is a core component of the mRNA decapping complex, a key factor in the regulation of mRNA decay. Focusing on these top GWAS findings, we next performed a number of different *in silico* and *in vitro* experiments to assess their potential role on miRNA function.

### *In silico* predictions of miRNA-to-mRNA binding and potential SNP effects

**Figure 1:**
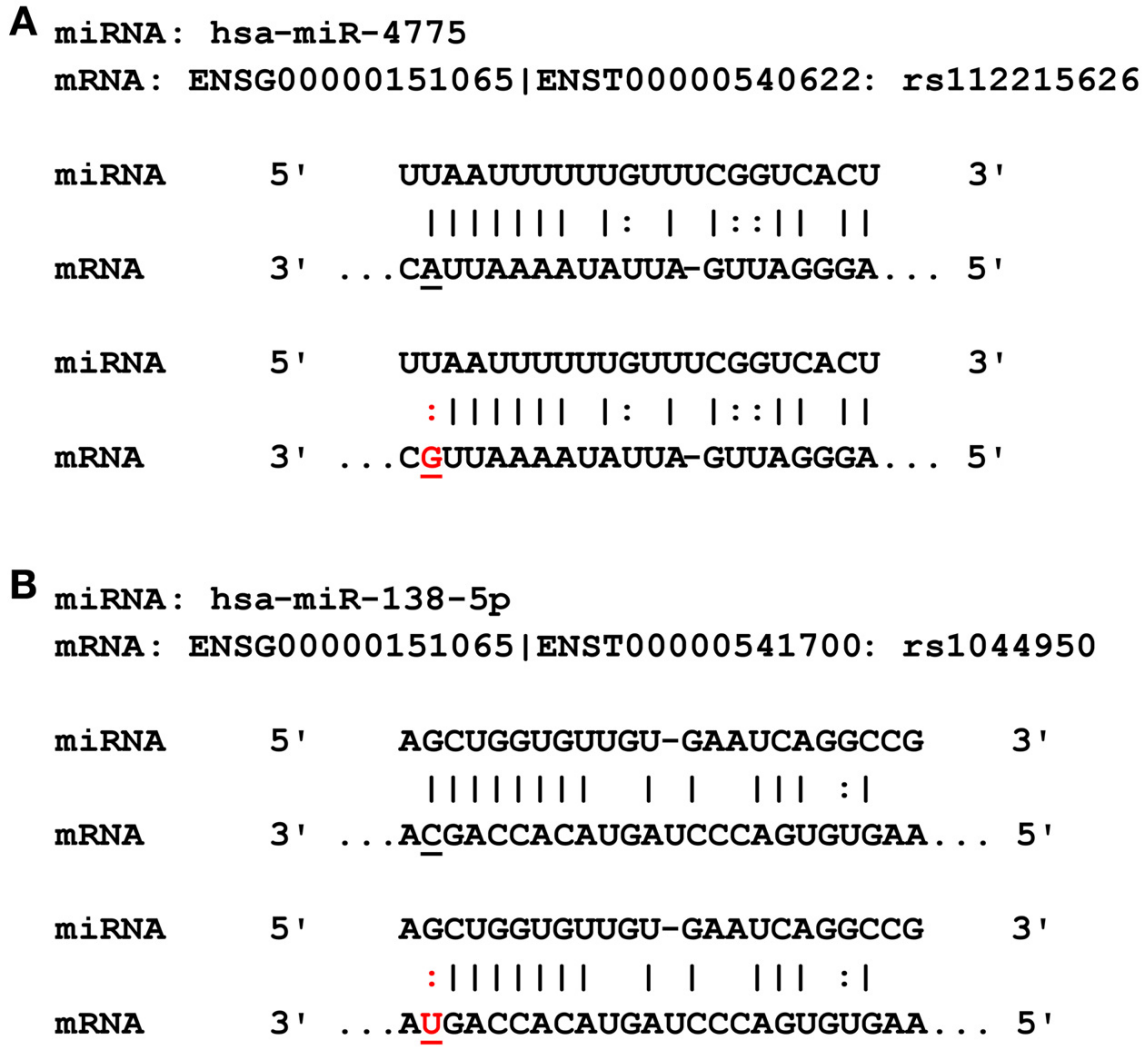
**Predicted miRNA binding sites in the *DCP1B* transcripts. (A)** Rs112215626 is located in the seed region of hsa-miR-4775. The alternative (G) allele of rs112215626 (highlighted in red) may alter binding affinity of hsa-miR-4775 to the *DCP1B* 3′UTR and therefore alter protein levels. **(B)** Rs1044950 is located in the seed region of hsa-miR-138-5p. The alternative T allele of the rs1044950 (highlighted in red) may alter binding affinity of hsa-miR-138-5p to the *DCP1B* 3′UTR and subsequently alter protein levels.

Using a bioinformatic tool recently developed in our group (Schilling, 2011, 2013), we predicted the potential role of the GWAS SNPs on chr 3p21.32, 8q13.3, and 12p13.33 [and their proxies, i.e., other SNPs in strong LD (*r*^2^ ≥ 0.5) and mapping within ±1 Mb] on their potential to interfere with miRNA-to-mRNA binding. These analyses did not identify any such effects for the GWAS signals on chr 3 or 8, but several potentially relevant predictions for the memory associated SNPs on chr 12p13.33 (Table 1). All of the SNPs predicted to interfere with miRNA binding were proxies of rs113948889 with *r*^2^ values of 1 and suggested a potential up-regulation of protein expression conferred by the respective non-reference alleles. The strongest effect was estimated for rs112215626, predicted to interfere with the binding of hsa-miR-4775 to the 3′UTR of *DCP1B* mRNA (see miRNA-to-mRNA alignment in Figure 1A). The second strongest effect was estimated for rs1044950, which was predicted to interfere with the binding of hsa-miR-138-5p to the 3′ UTR of another *DCP1B* transcript (Figure 1B). Interestingly, the same SNP is located within the coding sequence of alternative *DCP1B* transcripts where it is predicted to elicit an amino acid change from alanine to valine at the respective residues (i.e., Ala273Val, Ala249Val, Ala375Val; Supplementary Table 2). However, this missense substitution is not predicted to significantly alter protein function in any of the affected *DCP1B* transcripts (Supplementary Table 3) *in silico* based on estimates from PolyPhen2 (Adzhubei et al., 2010) or SIFT (URL: http://sift.bii.a-star.edu.sg/), indicating that the presumed effects on miRNA-to-mRNA binding may represent the overarching functional mechanism underlying the GWAS signal at this locus. Hence, we selected to follow-up the two leading potential *DCP1B* miRNA SNPs (i.e., rs112215626 and rs1044950) *in vitro*.

**Table 1:** **SNPs in LD with memory-associated SNP rs113948889 and predicted to affect miRNA-to-mRNA binding *in silico***.

**GWAS SNP**	**miRNA target site SNP**	**CHR**	**BP**	**Distance to GWAS SNP**	**miRNA target site SNP alleles**	**LD R2**	**microRNA**	**Target gene**	**Target transcript**
rs113948889	rs112215626	12	2,059,337	44,833	G/A	1	hsa-miR-4775	DCP1B	ENST00000540622
rs113948889	rs1044950	12	2,061,982	42,188	T/C	1	hsa-miR-138-5p	DCP1B	ENST00000541700
rs113948889	rs34730825	12	2,064,602	39,568	C/T	1	hsa-miR-3147	DCP1B	ENST00000543381
rs113948889	rs111963484	12	2,102,086	2,084	C/A	1	hsa-miR-4270	DCP1B	ENST00000535873
rs113948889	rs111963484	12	2,102,086	2,084	C/A	1	hsa-miR-4441	DCP1B	ENST00000535873
rs113948889	rs111963484	12	2,102,086	2,084	C/A	1	hsa-miR-505-5p	DCP1B	ENST00000535873
rs113948889	rs112637373	12	2,102,271	99	G/T	1	hsa-miR-2909	DCP1B	ENST00000535873
rs113948889	rs34730825	12	2,064,602	39,568	C/T	1	hsa-miR-92a-1-5p	DCP1B	ENST00000543381

*Legend: Predicted miRNA target site SNPs in linkage disequilibrium with GWAS SNP rs113948889 listed in descending order of predicted effect on miRNA-to-mRNA binding. Minor alleles are listed first in “SNP alleles” column. CHR, chromosome; BP, base-pair position on CHR; LD, linkage disequilibrium. Genomic annotations based on human genome (hg) build 19, transcript IDs according to ENSEMBL v.75. Shaded entries highlight SNPs assessed in vitro*.

### *In vitro* assessment of SNPs predicted to affect miRNA-to-mRNA binding

**Figure 2:**
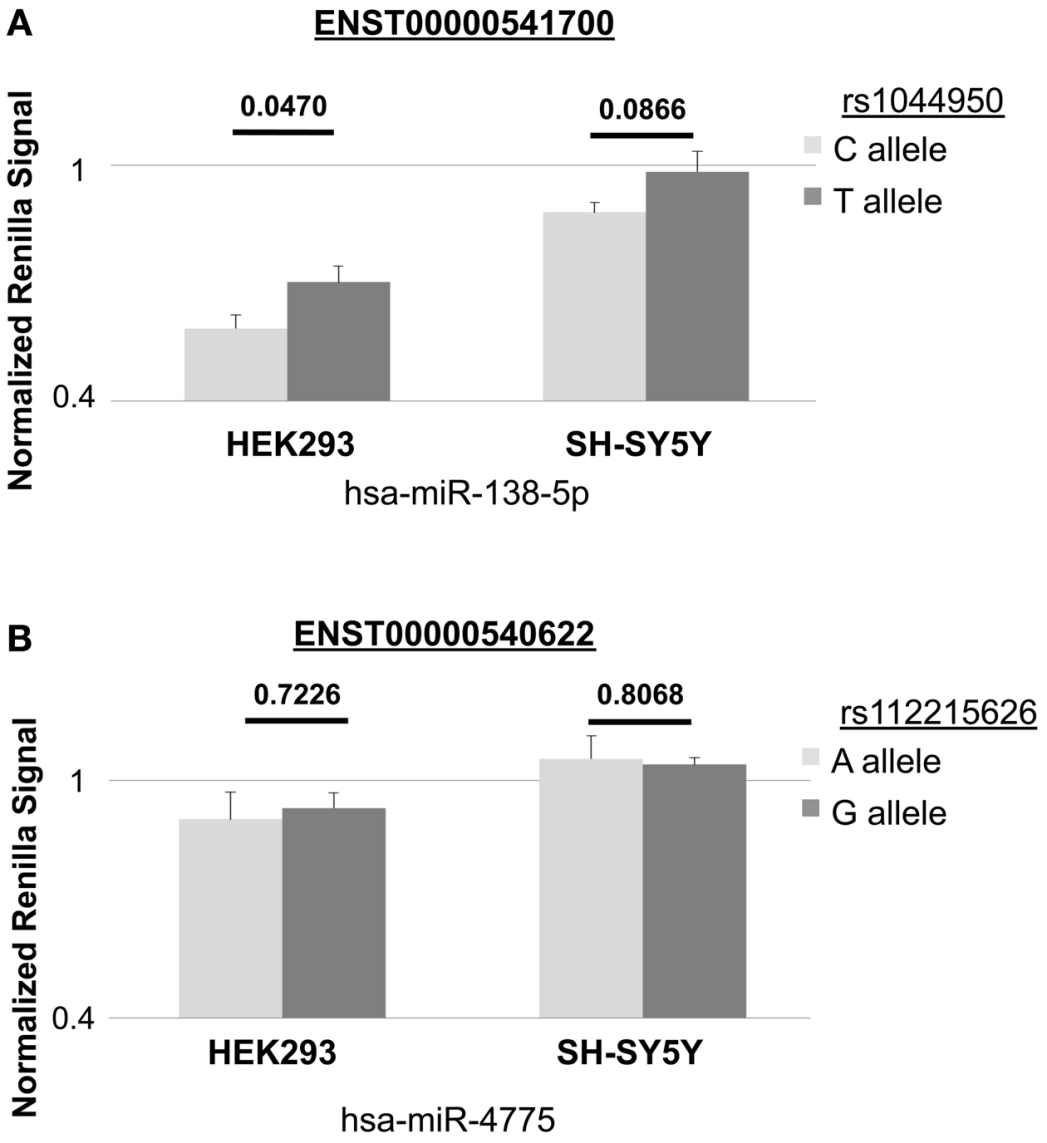
***In vitro* effects of rs1044950 and rs112215262 on miRNA-to-mRNA binding and gene expression**. The bar charts show the normalized *Renilla* luciferase expression in constructs containing *DCP1B* 3′UTR and corresponding SNP alleles. Depicted are the mean *Renilla* luciferase intensities and the standard errors relative to the control luciferase intensities of the construct co-transfected with the non-targeting miRNA control (corresponding to the horizontal line): **(A)** for transcript ENST00000541700 containing the reference (G) or alternative (A) allele of rs1044950 and co-transfected with has-miR-138-5p into HEK293 and SH-SY5Y cells. The relative mean luciferase luminescence of the construct containing the G and the A allele was 0.585 (±0.0335) and 0.703 (±0.0417) in HEK293 cells, and 0.880 (±0.0256) and 0.985 (±0.0513) in SH-SY5Y cells. **(B)** for transcript ENST00000540622 containing the reference (T) or alternative (C) allele of rs112215626 and co-transfected with hsa-miR-4775 into HEK293 and SH-SY5Y cells. The relative mean luciferase luminescence of the construct containing T and the C allele was 0.903 (±0.0692) and 0.931 (±0.0382) in HEK293 cells, and 1.056 (±0.0582) and 1.041 (±0.0185) in SH-SY5Y cells.

We conducted luciferase reporter assays in naïve HEK293 cells and SH-SY5Y cells using two different 3′UTR constructs (containing either the reference or non-reference alleles) of *DCP1B* transcripts ENST00000540622 and ENST00000541700 predicted to bind hsa-mir-138-5p and hsa-mir-4775, respectively. In HEK293 cells, co-transfections of the resulting 3′UTR *Renilla* vectors and hsa-mir-138-5p showed significant reductions of luciferase luminescence in comparison to co-transfections with the non-binding miRNA control (*P_one−tailed_* ≤ 0.000191), confirming our predictions that this miRNA binds to the corresponding 3′UTR *in vitro* (Figure 2A). Analyses comparing luciferase luminescence with respect to allele status at rs1044950 revealed a consistent and significant increase in normalized luciferase expression in constructs containing the minor A-allele (*n* = 7, *P* = 0.0470; Figure 2A). While co-transfection of the same reporter constructs with hsa-mir-138-5p to SH-SY5Y cells generally showed similar effects pointing in the same direction, the difference in luciferase expression between constructs containing the G- vs. A-allele was not statistically significant (*n* = 9, *P* = 0.0866, Figure 2A). Thus, in both cell lines the non-reference (minor) A-allele of SNP rs1044950 increased luciferase expression as compared to the reference (major) G-allele by 11.8% (HEK293) and 10.5% (SH-SY5Y). These findings are in line with our *in silico* predictions, which suggested stronger binding of hsa-mir-138-5p in 3′UTR sequences containing the reference (G) allele compared to the alternative (A) allele. In contrast, we did not observe a significant reduction of *Renilla* expression upon co-transfecting either reporter construct containing *DCP1B* transcript ENST00000540622 with SNP rs112215626 and hsa-mir-4775 compared to the non-binding miRNA in control experiments in neither HEK293 nor SH-SY5Y cells (*n* = 7 and 6, respectively, *P_one−tailed_* ≥ 0.05, Figure 2B). This suggests that this miRNA, as opposed to hsa-mir-138-5p, does not bind to the corresponding *DCP1B* transcript, at least under these experimental conditions. In summary, the results of the luciferase reporter experiments suggest that SNP rs1044950 elicits allele-specific effects on the expression of constructs containing the corresponding *DCP1B* 3′UTR in the presence of hsa-mir-138-5p *in vitro*.

### Analysis of expression levels of hsa-miR-138-5p and *DCP1B* mRNA in human post-mortem brain tissue

**Figure 3:**
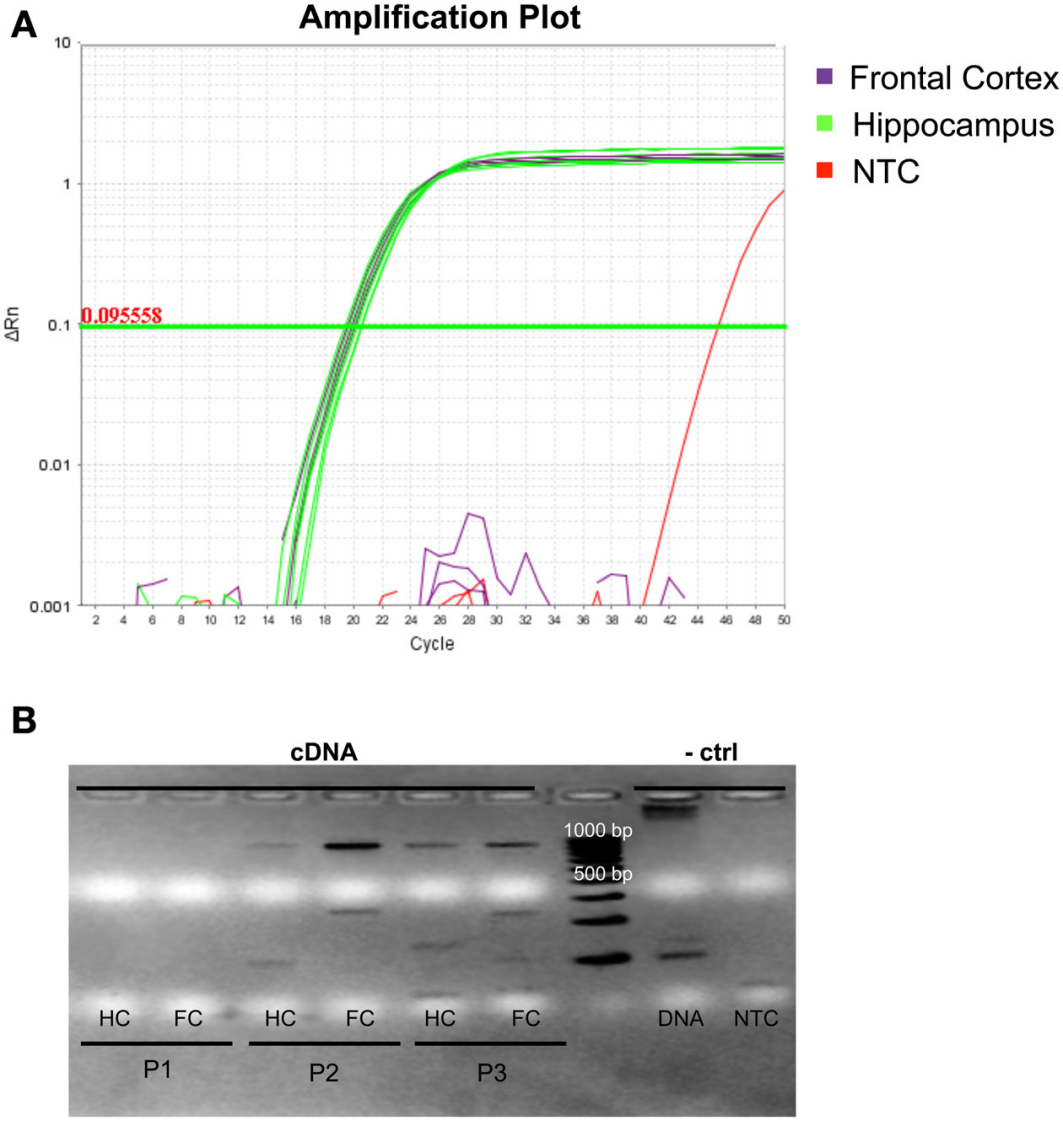
**Expression profile of hsa-miR-138-5p and *DCP1B* in autopsy brain tissues of three deceased human probands. (A)** Amplification plot of qPCR experiments in three frontal cortices (highlighted in purple) and three hippocampi (highlighted in green). Hsa-miR-138-5p was expressed in both frontal cortex (FC) and hippocampus (HC; C_*T*_ ~ 19). One of two NTCs (non-template control, highlighted in red) showed amplification at C_*T*_ > 45, whereas the other NTC did not show any amplification. **(B)** Ethidium bromide stained gel electrophoresis in 1% agarose displays the semi-quantitative levels of *DCP1B* cDNA (expected and observed at ~850 bp) in post-mortem human brain tissues. No expression was detected in proband 1 (P1). The *DCP1B* cDNA levels were higher in the FC, compared to HC of proband 2 (P2) and proband 3 (P3). **(A)** gDNA amplicon band (expected and observed at ~6.9 kb) could be detected and no band in the NTC.

While both hsa-mir-138-1 (Landgraf et al., 2007) and transcripts of *DCP1B* (URL: http://human.brain-map.org/) were previously shown to be expressed in both hippocampus and frontal cortex in humans, no data exist as to whether this expression actually occurs at the same time, a prerequisite condition for the interaction effects observed *in vitro* to also be relevant *in vivo*. To address this question, we obtained three different post-mortem hippocampus and frontal cortex specimen, which were profiled for both miRNA and mRNA expression patterns. qPCR of hsa-mir-138-5p revealed high expression of this miRNA in both hippocampal and frontal brain slices for all three individuals (Figure 3A). Hsa-mir-138-5p expression levels were comparable to those observed for hsa-miR-let-7b, a miRNA ubiquitously expressed in many human tissues including brain (and used here as positive control miRNA), in the same specimen (data not shown). Semi-quantitative PCR of *DCP1B* mRNA in the same brain samples revealed a more variable expression pattern (Figure 3B). Using this method *DCP1B* mRNA was only detectable in two of the three human brain samples (probands 2 and 3 in Figure 3B). Further, in both of these probands *DCP1B* mRNA expression levels were higher in frontal cortex as compared to hippocampus (owing to the semi-quantitative nature of the experiments, these differences could not be assessed for statistical significance). Regardless of the observed interindividual and regional expression differences, these experiments clearly demonstrate that both hsa-mir-138-5p and *DCP1B* mRNA are co-expressed simultaneously in both the hippocampi and frontal cortices in human brain, setting the temporal and spatial stage for an interaction of these two RNA molecules *in vivo*.

### Expression quantitative trait locus (eQTL) analyses of hsa-miR-138-5p using next-generation sequencing data

**Figure 4:**
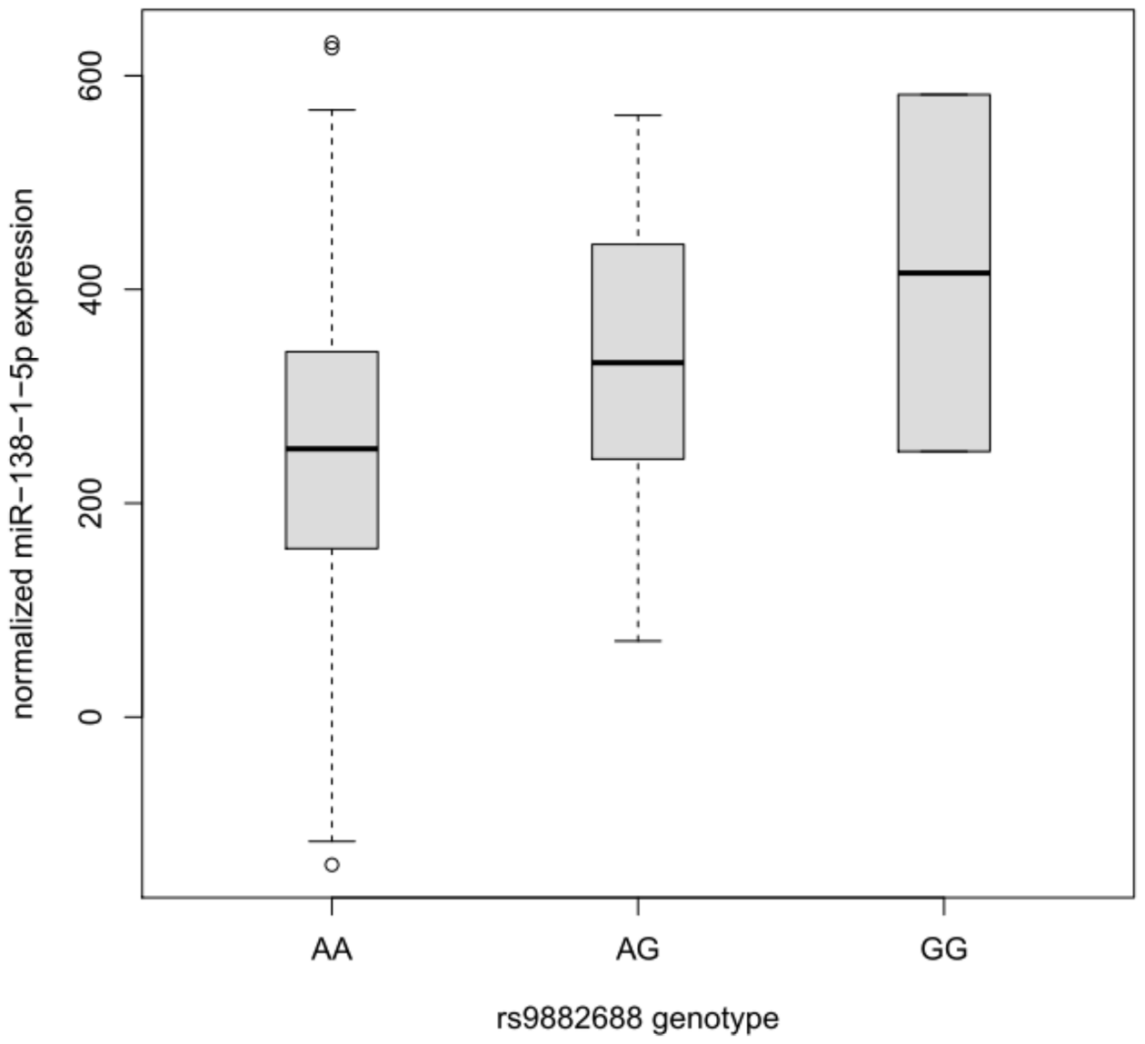
**Box plot of the distribution of hsa-miR-138-1-5p expression levels in lymphoblastoid cell lines dependent on the rs9882688 genotype in 308 individuals of European descent**. Horizontal lines represent median values, boxes represent interquartile ranges, and whiskers extend to 1.5× the interquartile range; values outside this range are depicted as circles. Carriers of the rs9882688 G allele showed a statistically significant (*P* = 0.000504) increase in hsa-miR-138-1-5p expression levels.

Although the lead GWAS signal (elicited by rs9882688) identified in this study was not predicted to interfere with miRNA-to-mRNA binding *in silico*, this SNP maps into a potential regulatory site 20 kb upstream of miRNA hsa-mir-138-1. The primary transcript of this miRNA is processed into two different mature products hsa-mir-138-5p and hsa-mir-138-1-3p, which are both expressed in humans (Landgraf et al., 2007). Notably, hsa-mir-138-5p is the same miRNA whose binding to *DCP1B* is also potentially affected by the presence of SNP rs1044950, as suggested by the *in silico* and *in vitro* experiments outlined above. Thus, a potential effect of rs9882688 on the expression of hsa-mir-138-5p could be indicative of a more systematic involvement of this miRNA in human memory performance. To this end, we performed eQTL analyses on NGS-based small-RNA sequencing data in over 300 human lymphoblastoid cell line samples from the GEUVADIS project (Lappalainen et al., 2013). The results of these analyses revealed a significant dose-dependent effect of this SNP on hsa-mir-138-5p expression in these peripheral cell lines. Specifically, the presence of the minor G-allele of rs9882688 was associated with increased hsa-miR-138-5p expression levels (*n* = 309, beta = 80.87, standard error [*SE*] = 23.00, *P*-value = 0.000504, Figure 4). This finding was not solely driven by the two G-allele homozygotes in this dataset as evidenced by eQTL results after exclusion of these individuals (*n* = 307, beta = 77.35, *SE* = 25.56, *P* = 0.00270). These data suggest that the same SNP showing association with episodic memory performance in humans, also significantly correlates with changes in hsa-mir-138-5p expression in human peripheral cell lines.

## Discussion

We investigated the potential role of common DNA sequence variants associated with human memory performance in miRNA-mediated regulation of gene expression. A GWAS analysis on 13 memory traits in up to 1318 individuals aged 60 years or older revealed a number of potential association signals in both working (rs113948889) and episodic memory (rs9882688 and rs1016365) domains. Among these were SNPs predicted to be involved in the expression and function of hsa-mir-138-5p, a miRNA known to be important in brain development and function. Specifically, genotypes at SNP rs9882688 (which represented the lead memory GWAS signal in this study) were shown to correlate significantly with the expression of this hsa-mir-138-5p in human LCLs using next-generation small-RNA sequencing data. In addition, we found that SNP rs1044950 (as proxy of another top GWAS signal) leads to allele-specific changes in the expression of reporter constructs containing the 3′UTR sequence of *DCP1B* transcripts predicted to contain binding sites for hsa-mir-138-5p in peripheral (HEK293, *P* = 0.047) and neuronal (SH-SY5Y, *P* = 0.0866) human cell lines. Finally, expression profiling of both hsa-mir-138-5p and *DCP1B* mRNA in post-mortem brain tissue revealed that both putative interactants are co-expressed in brain. In summary, in this study various lines of independent evidence converge on the notion that hsa-mir-138-5p may play a significant role in processes related to human episodic memory performance.

The data from our study are in line with previous research on the potential role of mir-138 in mammalian brain function. For instance, Miska (Miska et al., 2004) showed that mir-138 expression levels increase with age in the developing rat brain reaching their peak in juvenile and adult rats. Landgraf (Landgraf et al., 2007) later showed that mir-138 was also highly expressed in adult human brain samples, including frontal cortex and hippocampus. More recently, mir-138 was identified as part of a functional screen for dendritic miRNAs that regulate spine morphogenesis in rats (Siegel et al., 2009). In that study, mir-138 was found to act as a negative regulator of dendritic spine size possibly by tuning the activity of antagonistic signaling that regulate the actin cytoskeleton in spines. In a review on the same topic Schratt hypothesized that miR-138-related pathways might also contribute to long-lasting forms of synaptic plasticity […]” (Schratt, 2009). Our data, in which we observed multiple converging lines of genetic evidence suggesting a potential role of hsa-mir-138-5p in episodic memory performance, provide some first independent support of this hypothesis and extend it to humans. In addition to *DCP1B*, hsa-mir-138-5p is predicted to target a large number of different human transcripts *in silico* (ranging between a few hundreds to a few thousands depending on the prediction algorithm used). In the GWAS results generated here, genes containing hsa-mir-138-5p targets identified by three or more of the prediction algorithms show a significant (*P* < 0.05 based on 100,000 permutations) enrichment for memory-associated SNPs as compared to genes not targeted by this miRNA (Supplementary Table 4), further supporting the notion that hsa-mir-138-5p represents a potential molecular regulator of human memory function.

The gene *DCP1B* encodes “decapping mRNA 1B” which is a core component of the mRNA decapping complex, a key factor in the regulation of mRNA decay. Decapping and mRNA degradation takes places in P-bodies (processing bodies) (Kulkarni et al., 2010). Importantly, P-bodies have been suggested to be one of the predominant sites of miRNA-mediated mRNA degradation, a process that is inhibited upon depletion of the decapping DCP1:DCP2 complex (Behm-Ansmant et al., 2006). Thus, *DCP1B* and mir-138 function within the same general pathway, i.e., the degradation of mRNAs bound by miRNAs, likely including those targeted by hsa-mir-138 itself.

While the novel experimental data generated here support the hypothesis that hsa-mir-138-5p plays an important role in physiological mechanisms involved in human memory performance, we note the following potential limitations of our findings. First and foremost, the functional genetic experiments implying a role of rs1044950 in interfering with miRNA-to-mRNA binding are based on *in vitro* experiments using reporter constructs. While it is tempting to speculate that these effects are also relevant *in vivo*, no direct experimental proof of this interpretation currently exists. In humans, however, this type of evidence is difficult to come by owing to the fact that molecular mechanisms in the living human brain cannot be monitored at sufficient resolution with current technologies. In order for the hypothesized effects to take place *in vivo*, both interactants need to be co-expressed in the same tissue at the same time. Our RNA-profiling experiments using human frontal cortex and hippocampus samples clearly demonstrate that this is the case for hsa-mir-138-5p and *DCP1B*. Second, another conclusion of this study is that SNP rs9882688 is involved in the regulation of hsa-mir-138-5p expression. While this could be shown in LCLs, it remains unclear whether these effects are also relevant in human brain. Third, linking the GWAS results for rs9882688 to this SNP's eQTL effects on hsa-mir-138-5p suggests that the same (i.e., G) allele increasing miRNA expression is associated with worse episodic memory performance (indicated by the negative beta-coefficient in Supplementary Table 1). Increased miRNA abundance is typically interpreted to lead to an increase in mRNA target repression and, as a consequence, reduced target gene expression. For SNP rs1044950, however, the minor (A) allele, associated with worse memory performance, leads to a decrease in miRNA-to-mRNA binding *in vitro*. This latter finding would suggest an increased target gene expression, i.e., an effect opposite to what can be expected from the eQTL results with rs9882688. One possible explanation for these different effect directions is the involvement of competing endogenous RNAs (ceRNAs). Such ceRNAs were recently proposed to exist, e.g., generated from transcribed pseudogenes, long noncoding RNAs, or stable RNAs (Salmena et al., 2011; Helwak et al., 2013). Here they could lead to increased levels of hsa-mir-138-5p in A-allele carriers of rs1044950, i.e., similar to what is observed for rs9882688-G. Finally, it needs to be emphasized that the GWAS results reported here represent preliminary findings that need to be replicated in independent data sets. However, even if these were to reveal smaller effect estimates than those reported here (e.g., as a result of the “winner's curse”; Kraft, 2008), this should have no bearing on the functional genetic and expression profiling results of our study. In addition to generating independent genetic association data, future work will need to extend our eQTL findings to the CNS, confirm the regulatory role of hsa-mir-138-5p on endogenous expression of *DCP1B* and other target genes and their corresponding proteins (in particular those potentially involved in human memory function, such as *WWC1* [*KIBRA*]), and assess the role of this miRNA on their putative targets *in vivo*.

In summary, by combining unbiased genome-wide screening with extensive *in silico* modeling, *in vitro* functional assays, and gene expression profiling, our study identified hsa-mir-138-5p as a potential molecular regulator of human memory function. Future work is needed to assess the relevance of these findings *in vivo* and to explore other regulators and targets of this highly abundant miRNA, in particular their connection to memory and other cognitive domains.

### Conflict of interest statement

The authors declare that the research was conducted in the absence of any commercial or financial relationships that could be construed as a potential conflict of interest.
