# Supplementary material for: MicroRNA-138 is a potential regulator of memory performance in humans
Source: Front Hum Neurosci. 2014 Jul 11;8:501. doi: 10.3389/fnhum.2014.00501 (PMC4093940; doi:10.3389/fnhum.2014.00501)

Suppl. Figure 2a: Manhattan plot of GWAS results for trait “WL_save”

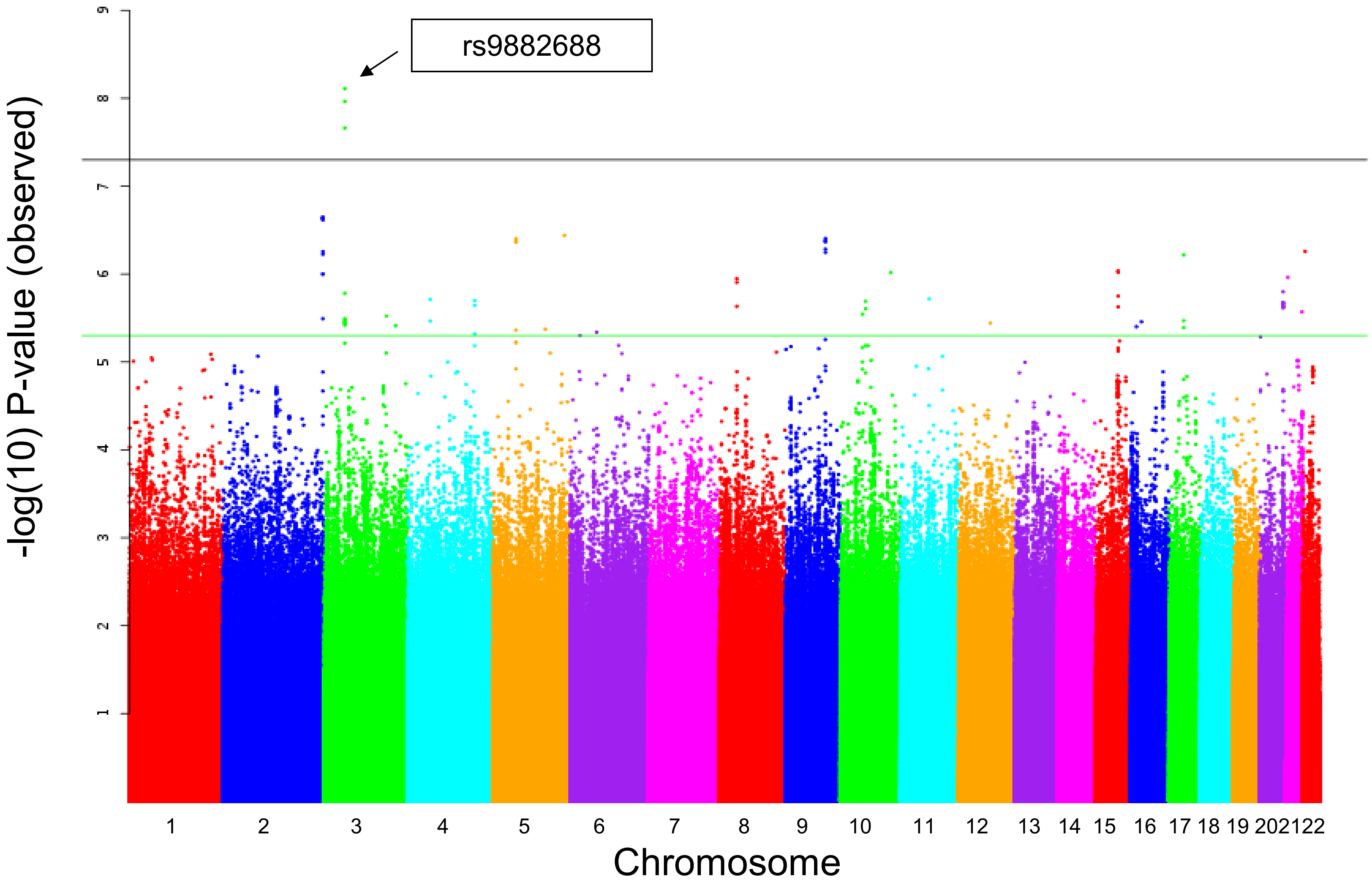

Suppl. Figure 2b: Q-Q plot of GWAS results for trait “WL_save” (lambda: 1.009)

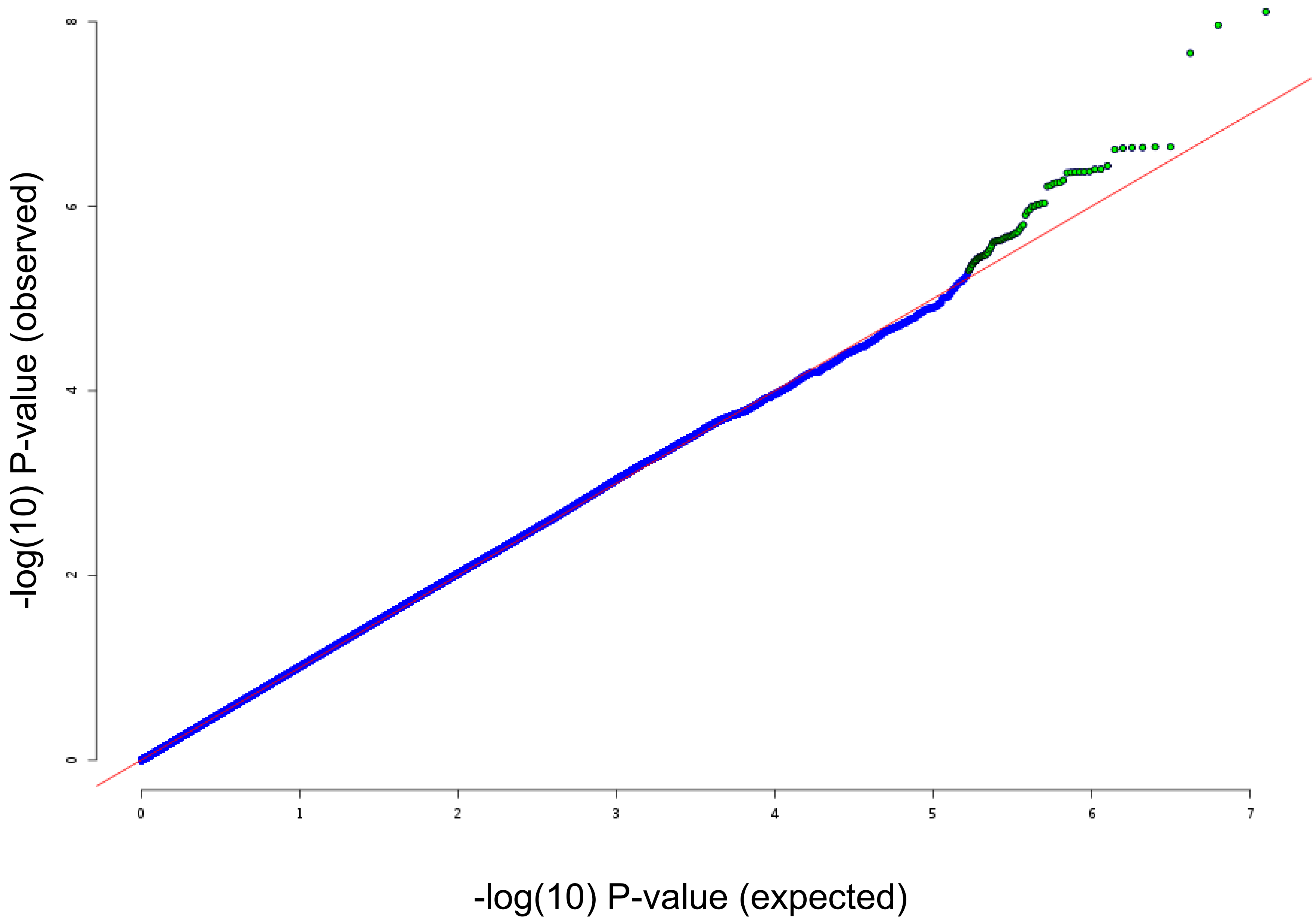

Supplement: Supplementary file 1 [file SupplementaryMaterial.zip › Presentation 1/Supplementary Figure 2.PDF]
