# Supplementary material for: MicroRNA-138 is a potential regulator of memory performance in humans
Source: Front Hum Neurosci. 2014 Jul 11;8:501. doi: 10.3389/fnhum.2014.00501 (PMC4093940; doi:10.3389/fnhum.2014.00501)

<u>Suppl. Figure 3a:</u> Manhattan plot of GWAS results for trait "ItemItem"

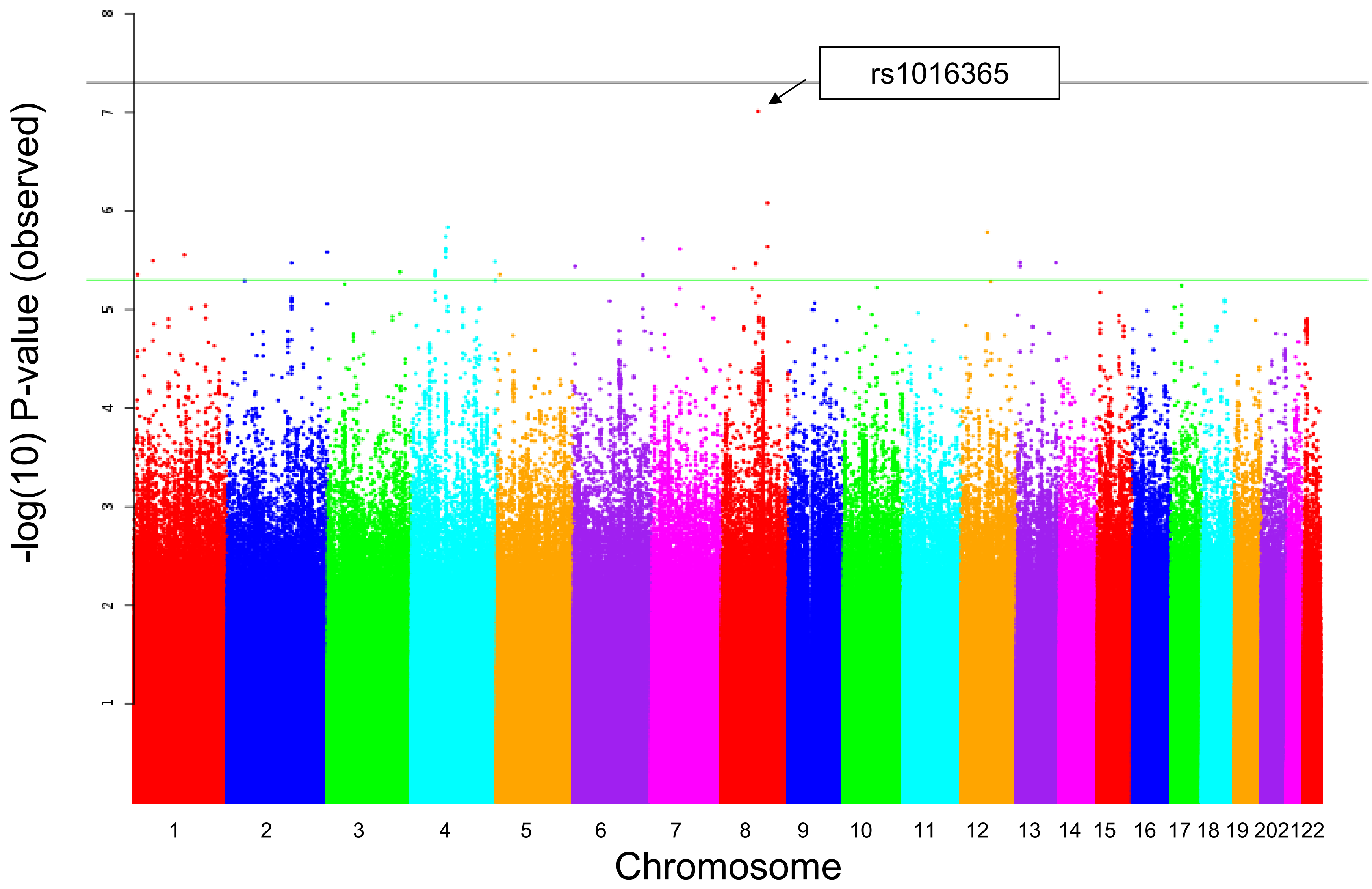

<u>Suppl. Figure 3b:</u> Q-Q plot of GWAS results for trait "ItemItem" (lambda: 1.009)

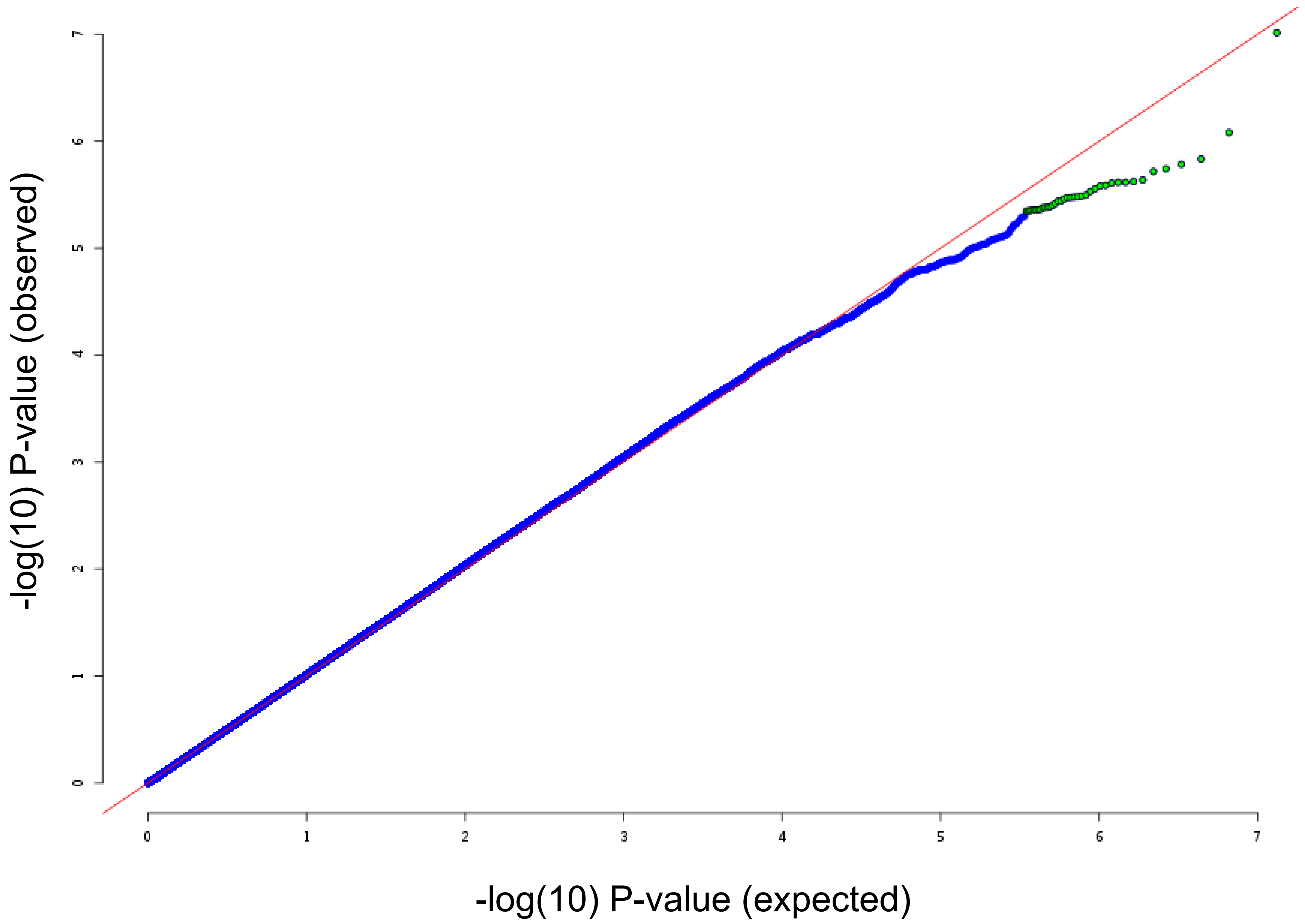

Supplement: Supplementary file 1 [file SupplementaryMaterial.zip › Presentation 1/Supplementary Figure 3.PDF]
