# Supplementary figures and images for: MicroRNA-138 is a potential regulator of memory performance in humans
Source: Front Hum Neurosci. 2014 Jul 11;8:501. doi: 10.3389/fnhum.2014.00501 (PMC4093940; doi:10.3389/fnhum.2014.00501)

Normal Q-Q Plot

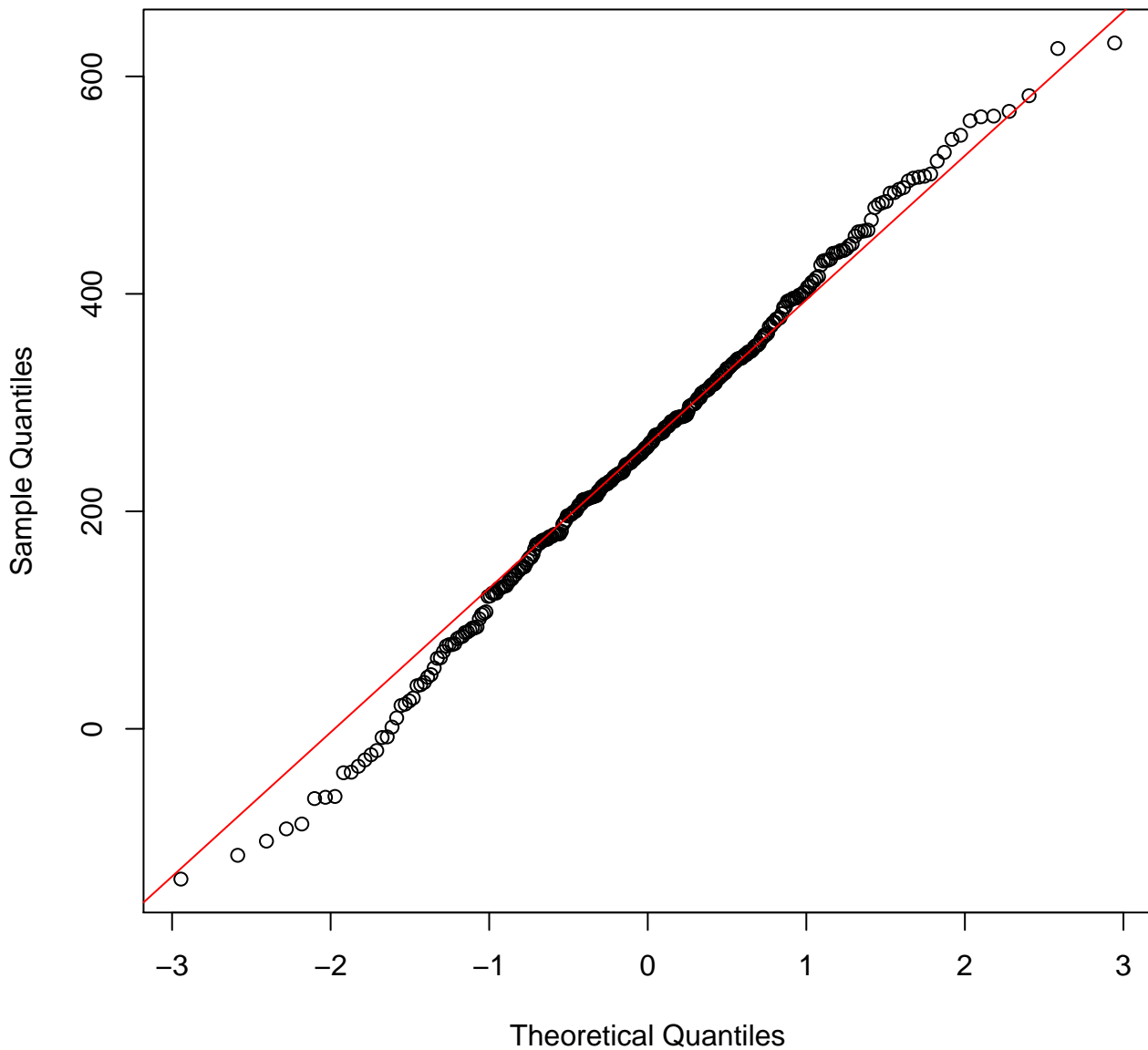

Supplement: Supplementary file 1 [file SupplementaryMaterial.zip › Presentation 1/Supplementary Figure 1.PDF]
