# Supplementary material for: MicroRNA-138 is a potential regulator of memory performance in humans
Source: Front Hum Neurosci. 2014 Jul 11;8:501. doi: 10.3389/fnhum.2014.00501 (PMC4093940; doi:10.3389/fnhum.2014.00501)
Supplement: Supplementary file 1 [file SupplementaryMaterial.zip › Presentation 1/Supplementary Methods_Table 1.DOC]

**MicroRNA-138 is a potential regulator of**

**memory performance in humans**

**Julia Schröder^1,2^, Sara Ansaloni^1^, Marcel Schilling^1,3^, Tian Liu^1,4^, Josefine Radke^5,^ Marian Jaedicke^1^, Brit-Maren M. Schjeide^1^,** Andriy V. Mashychev^1^, Christina Tegeler^2^, **Helena Radbruch^5^, Goran Papenberg^4,6^, Sandra Düzel^4^, Ilja Demuth^2,7^, Nina Bucholtz^2^, Ulman Lindenberger^4^, Shu-Chen Li^4,8^, Elisabeth Steinhagen-Thiessen^2^, Christina M. Lill^1,9^, Lars Bertram^1,10*^**

**^1^Department of Vertebrate Genomics, Max Planck Institute for Molecular Genetics. Berlin, Germany**

**^2^Charité Research Group on Geriatrics, Charité – Universitätsmedizin Berlin, Berlin, Germany**

**^3^**Berlin Institute for Medical Systems Biology, **Max Delbrück Center for Molecular Medicine, Berlin, Germany**

**^4^Center for Lifespan Psychology, Max Planck Institute for Human Development, Berlin, Germany**

**^5^Department of Neuropathology, Charité – Universitätsmedizin Berlin, Berlin, Germany**

**^6^Aging Research Center, Karolinska Institute, Stockholm, Sweden**

**^7^Institute of Medical and Human Genetics, Charité – Universitätsmedizin Berlin, Berlin, Germany**

**^8^Department of Psychology, Lifespan Developmental Neuroscience, TU Dresden, Dresden, Germany**

**^9^**Department of Neurology, Focus Program Translational Neuroscience, University Medical Center of the Johannes Gutenberg University Mainz, Mainz, Germany

**^10^**School of Public Health, Faculty of Medicine, Imperial College London, London, UK

**No. of figures: 4**

**Supplementary Methods**

*Details on cognitive testing procedures assessing memory performance.* For the genetic association analyses of this study, we used a total of 13 quantitative measures of episodic memory and working memory derived from: (a) the Consortium to Establish a Registry for Alzheimer’s Disease (CERAD) cognitive battery (Morris *et al.* 1989) on word list memory and word list recall; (b) forward and backward serial recall paradigms (Li *et al.* 2010, Li *et al.* 2013); (c) associative memory tasks that assessed item memory as well as item-pair recognition (Preuschhof *et al.* 2010); and (d) an image recognition test at retention intervals of 2.5 hours and one week (Papenberg *et al.* 2011). Below we describe all non-CERAD related procedures in more detail. Information on the CERAD cognitive test battery can be found in Morris *et al.* (1989) and URL: <http://www.memoryclinic.ch/content/view/37/47/>). See also Supplementary Table 1 for a summary of cognitive assessments.

1. Forward and backward serial recall task. For this task, participants were presented with six different lists of 12 words each. After the presentation of the last item in each list, the participants were asked to recall each word at its correct position. Word Lists 1 to 3 were recalled in forward order (from the first word to the last word of the list), whereas Word Lists 4 to 6 were recalled in backward order. Recall was self-paced. For each list, responses were scored using a strict serial-recall criterion: an accurate response required that both the identity of a word and its serial position were correct.

2. Item and pair associative episodic memory task. The item and pair associative episodic memory task (henceforth labeled as the “item-pair” task) had four conditions that were tested sequentially in one session. During an initial study phase, participants were visually presented with pairs of unrelated words and were instructed to study each pair under two conditions: either as two single words (item instruction) or as a pair of words (pair instruction). The study phase of each condition contained 30 pairs of semantically unrelated words. In the test phrase, subjects in one condition (item recognition) were asked whether they had seen the presented word during the study phase. Half of the words were old (target items), and the other half were new (distractor items). In the second condition (associative recognition), subjects had to decide whether a presented pair of words had been presented during the study phase. Half of the presented word pairs were old (target pairs), and the others were formed by recombining words in the previously studied lists (rearranged distractor pairs). Taken together, by crossing over the two instruction conditions with the two test conditions, the task resulted in four conditions that assessed item memory (item–item and pair-item tests) and associative memory (pair-pair and item-pair tests). Recognition memory performance was measured as hits minus false alarms to minimize the effects of potential individual differences in response bias.

3. Image recognition memory with retention. Performance in the image recognition memory task was assessed at two retention intervals: 2.5 hours and one week. At the beginning of the first session, participants were presented with 48 complex, colored images of scenes of neutral emotional valence; all were derived from the International Affective Picture System (Lang *et al*. 1997). The images were encoded incidentally: during the study phase, participants were required to determine whether the scene was “indoor” or “outdoor”—there were 24 scenes in each category—without explicit requirement of memorization. During retrieval, participants viewed each image for 3 seconds and were asked to determine whether each scene had been presented (“old”) or not presented (“new) during encoding. In each retrieval test, 24 unique old scenes and 24 unique new scenes (lures) were presented. Taking response bias into account, memory performance was measured as hits minus false alarms.

*Genome-wide SNP genotyping, quality control and imputation procedures.* DNA from all BASE-II participants was extracted from whole blood using standard procedures and then subjected to microarray-based SNP genotyping using the "Genome-Wide Human SNP Array 6.0" (Affymetrix, Inc.), according to the manufacturer’s instructions. Prior to imputation (see below), we applied the following quality control (QC) filters which led to an exclusion of SNPs that 1) violated Hardy-Weinberg equilibrium (HWE) at p ≤ 1e−6, and 2) showed call rate less than 98%. This filtering resulted in 829,344 autosomal SNPs in 1,946 BASE-II participants. Of these individuals, 214 were excluded from subsequent analysis because 1) they were missing information on “age” or “years of education” (used as co-variates in the linear regression model, see below), 2) showed call rates <95%, 3) showed evidence for sample duplication, relatedness or contamination, 4) showed inconsistencies between recorded and genotypic sex, 5) showed excessive heterozygosity, or 7) represented population outliers. The latter was determined using the EIGENSOFT program (Price *et al.* 2006). As all samples in our study were of self-reported Caucasian descent, we excluded ethnic outliers using the “Eigenstrat” function in EIGENSOFT with iterative outlier removal. After the above filtering steps, principal components (PC) were computed again for the 1,732 remaining samples. Based on examination of the scree plot, the first three PCs were retained and used as covariates during the association analysis in order to adjust for potential residual population stratification. Genome-wide imputation of unobserved genotypes was carried out on the QC'ed data using the IMPUTE2 [v2.2.2] software (Marchini *et al.* 2007) based on precompiled "ALL 1000G phase1interim" reference panels from the IMPUTE website (June 2011 release). As suggested by Southam *et al.* (2011) ensure an appropriate level of quality for imputed SNPs, we also applied post-imputation QC filtering including only SNPs with IMPUTE-information thresholds larger than or equal to 0.8 and minor allele frequencies at or above 5%. After this post-imputation filtering, a total of 5,371,945 high-quality SNPs were retained for all subsequent association analyses.

**References for Supplementary Methods**

Guerrero-Berroa, E., Ravona-Springer, R., Schmeidler, J., Silverman, J.M., Sano, M., Koifmann, K., Preiss, R., Hoffman, H., Heymann, A., Schnaider Beeri, M. (2014). Age, gender, and education are associated with cognitive performance in an older Israeli sample with type 2 diabetes. Int J Geriatr Psychiatry 29 (3), 299-309.

Lang PJ, Bradley, MM, Cuthbert, BN. International affective picture system (IPAS): Technical manual and affective ratings. Gainesville, FL: NIMH Center for the Study of Emotion and Attention. University of Florida, 1997.

Li, S.C., Chicherio, C., Nyberg, L., von Oertzen, T., Nagel, I.E., Papenberg, G., Sander, T., Heekeren, H.R., Lindenberger, U., Bäckman, L. (2010). Ebbinghaus revisited: Influences of the BDNF Val66Met polymorphism on backward serial recall are modulated by human aging. J Cogn Neurosci 22 (10), 2164-73.

Li, S.C., Papenberg, G., Nagel, I.E., Preuschhof, C., Schröder, J., Nietfeld, W., Bertram, L., Heekeren, H.R., Lindenberger, U. Bäckman, L. (2013). Aging magnifies the effects of dopamine transporter and D2 receptor genes on backward serial memory. Neurobiol Aging 34 (1) 358.e1-10.

Marchini, J., Howie, B., Myers, S., Mcvean, G., Donnelly, P. (2007). A new multipoint method for genome-wide association studies by imputation of genotypes. Nat Genet 39 (7), 906-13.

Morris, J.C., Heyman, A., Mohs, R.C., Hughes, J.P., van Belle, G., Fillenbaum, G., Mellits, E.D., Clark, C. (1989). The Consortium to Establish a Registry for Alzheimer's Disease (CERAD). Part I. Clinical and neuropsychological assessment of Alzheimer's disease. Neurology 39 (9), 1159-65.

Nagel, I.E., Chicherio, C., Li, S.C., Von Oertzen, T., Sander, T., Villringer, A., Heekeren, H.R., Bäckman, L., Lindenberger, U. (2008). Human aging magnifies genetic effects on executive functioning and working memory. Front Hum Neurosci 2, 1.

Papenberg, G., Bäckman, L., Chicherio, C., Nagel, I.E., Heekeren, H.R., Lindenberger, U., Li, S.C. (2011). Higher intraindividual variability is associated with more forgetting and dedifferentiated memory functions in old age. Neuropsychologia 49 (7), 1879-88.

Preuschhof, C., Heekeren, H.R., Li, S.C., Sander, T., Lindenberger, U., Bäckman, L. (2010). KIBRA and CLSTN polymorphisms exert interactive effects on human episodic memory. Neuropsychologia 48 (2), 402-8.

Price, A.L., Patterson, N.J., Plenge, R.M., Weinblatt, M.E., Shadick, N.A., Reich, D. (2006). Principal components analysis corrects for stratification in genome-wide association studies. Nat Genet 38 (8), 904-9.

Southam, L., Panoutsopoulou, K., Rayner, N.W., Chapman, K., Durrant, C., Ferreira, T., Arden, N., Carr, A., Deloukas, P., Doherty, M., Loughlin, J., McCaskie, A., Ollier, W.E., Ralston, S., Spector, T.D., Valdes, A.M., Wallis, G.A., Wilkinson, J.M., Arc, O.C., Marchini, J., Zeggini, E. (2011). The effect of genome-wide association scan quality control on imputation outcome for common variants. Eur J Hum Genet 19 (5), 610-4.

**Supplement Figures Legends**

**Supplement Figure 1:** Quantile-quantile plot of the hsa-miR-138-1-5p expression levels in peripheral blood mononuclear cells across 308 individuals of European descent.

**Supplement Figure 2:** *(a)* Manhattan plot of GWAS results for trait “WL_Save”. The SNP rs9882688 (P-value: 7.8 x 10^-9^) on human chromosome 3 is located ~20kb upstream of hsa-miR-138-1. *(b)* Quantile-quantile plot of the GWAS results for trait “WL-save”.

**Supplement Figure 3:** *(a)* Manhattan plot of GWAS results for trait “ItemItem”. The SNP rs1016365 (P-value: **9.7 x 10^-8^**) on human chromosome 8 is located ~6kb downstream of *EYA1* (**Homo sapiens eyes absent homolog 1 [Drosophila])**. *(b)* Quantile-quantile plot of the GWAS results for trait “ItemItem”.

**Supplement Figure 4:** *(a)* Manhattan plot of GWAS results for trait “TFEUWC”. The SNP rs113948889 (P-value: 9.9 x 10^-8^) on human chromosome 12 is located in an intron of *DCP1B* (**encoding decapping mRNA 1B)**. *(b)* Quantile-quantile plot of the GWAS results for trait “TFEUWC”.

**Supplementary Table 1:** Summary of cognitive measures / traits assessed by genome-wide association screening.

| **Domain** | **Test (abbrev)** | **Description** | **Reference for details** |
| --- | --- | --- | --- |
| WM | TCSWM | T-score of spatial working memory | Nagel *et al.* (2008), Papenberg et al. 2011) |
| WM | TFEUWC | T-score of spatial working memory combined with tasks testing frontal executive control (here: Wisconsin Card Sorting Test) | Nagel *et al.* (2008) |
| EM | CERAD_WL_learn | CERAD battery: Word list learning | Guerrero-Berroa *et al.* (2014) |
| EM | CERAD_WL_recall | CERAD battery: Word list recall | Guerrero-Berroa *et al.* (2014) |
| EM | CERAD_WL_save | Proportion of learned words vs. recalled words (max. 100%) | Guerrero-Berroa *et al.* (2014) |
| EM | Item_Item | Item instruction & item test (proportion of hits minus false alarms) | Preuschhof *et al.* (2010) |
| EM | Item_Pair | Item instruction & associative test (proportion of hits minus false alarms) | Preuschhof *et al.* (2010) |
| EM | Pair_Pair | Pair instruction & associative test (proportion of hits minus false alarms) | Preuschhof *et al.* (2010) |
| EM | SRFO | Serial recall: accuracy of forward recall | Li *et al.* (2010), Papenberg *et al.* (2011), Li *et al.* (2012) |
| EM | SRBK | Serial recall: accuracy of backward recall | Li *et al.* (2010), Papenberg *et al.* (2011), Li *et al.* (2012) |
| EM | Delayed_recall | Image recognition after 2.5 hours (proportions of hits minus false alarms) | Papenberg *et al.* (2011) |
| EM | Delayed_recall | Image recognition after 1 week (proportions of hits minus false alarms) | Papenberg *et al.* (2011) |

Abbreviations: WM = working memory; EM = episodic memory; CERAD = Consortium to Establish a Registry for Alzheimer’s Disease.
